# AImedReport: A Prototype Tool to Facilitate Research Reporting and Translation of Artificial Intelligence Technologies in Health Care

**DOI:** 10.1016/j.mcpdig.2024.03.008

**Published:** 2024-04-06

**Authors:** Tracey A. Brereton, Momin M. Malik, Lauren M. Rost, Joshua W. Ohde, Lu Zheng, Kristelle A. Jose, Kevin J. Peterson, David Vidal, Mark A. Lifson, Joe Melnick, Bryce Flor, Jason D. Greenwood, Kyle Fisher, Shauna M. Overgaard

**Affiliations:** a Center for Digital Health, Mayo Clinic, Rochester, MN; b Department of Family Medicine, Mayo Clinic, Rochester, MN

The core of artificial intelligence (AI) research in health care is carried out by AI data scientists, AI engineers, and clinicians; however, successfully evaluating and translating AI technologies into health care requires cross-collaboration beyond this group. Throughout ideation, development, and validation, successful translation requires engaging with many domains, including AI ethicists, quality management professionals, systems engineers, and more.^1^, ^2^, ^3^, ^4^, ^5^ We found through a scoping review that the prioritization of proactive evaluation of AI technologies, multidisciplinary collaboration, and adherence to investigation and validation protocols, transparency and traceability requirements, and guiding standards and frameworks are expected to help address present barriers to translation.^6^ However, as identified by Lu et al^7^ through a systematic review assessing clinical prediction model adherence to reporting guidelines that no consensus exists regarding model details that are essential to report, with some reporting items being commonly requested across reporting guidelines yet other reporting items being unique to specific reporting guidelines. Unless there is clear, consistent, and unified best practices and communication and collaboration across domains, there will be gaps in development, accountability, and implementation.^6^, ^7^, ^8^, ^9^, ^10^ Documentation is a crucial part of reporting and translation, but its coordinated maintenance throughout the AI lifecycle remains a challenge.^6^^,^^9^, ^10^, ^11^

We have established a proof-of-concept team-based documentation strategy for AI translation to simplify compliance with evaluation and research reporting standards through the development of AImedReport, a reporting guideline documentation repository (Figure). AImedReport organizes available reporting guidelines for different phases of the AI lifecycle, consolidating reporting items from different guidelines, assigning specific roles to team members, and guiding relevant information to capture when knowledge is generated (Appendix A).

**Figure:**
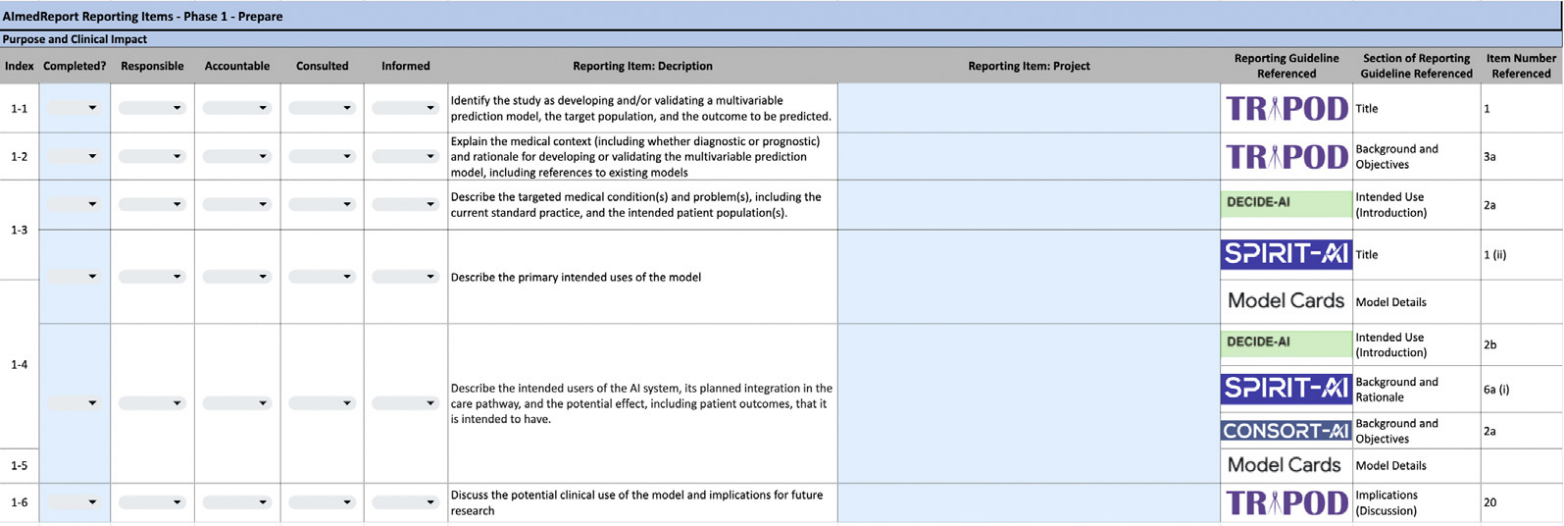
Prepare phase of AImedReport.

### Method of Development

We established a centralized documentation repository by first conducting a scoping review^6^ to investigate and understand the existing landscape of AI documentation and available resources (eg, reporting guidelines, protocols, standards, and frameworks). Within the scoping review, we found that documentation resources were fragmented throughout several reporting guidelines, prompting the consolidation and organization of such resources into AImedReport as a tool to structure available reporting guidelines in accordance with the AI lifecycle, reduce repetitive documentation burden, and promote knowledge continuity. Six research reporting guidelines make up the AImedReport: CONSORT-AI,^12^ DECIDE-AI,^13^ ML Test Score,^14^ Model Card,^15^ SPIRIT-AI,^16^ and TRIPOD^17^ (Table 1). The items that make up each reporting guideline are included in the AImedReport as “Reporting Items” and describe considerations for teams to document and maintain.

**Table 1:** Description of Reporting Guidelines Included Within the AImedReport

Reporting guideline	Description
Consolidated Standards of Reporting Trials—Artificial Intelligence (CONSORT-AI)	Aims to promote transparency and completeness in reporting clinical trials for AI interventions, helping to understand, interpret, and appraise the quality of clinical trial design and risk of bias in the reported outcomes; focuses on reporting the results of clinical trials
Developmental and Exploratory Clinical Investigation of Decision-support systems driven by Artificial Intelligence (DECIDE-AI)	Aims to improve the reporting of studies describing the evaluation of AI-based decision-support systems during their early, small-scale implementation in live clinical settings
ML Test Score	Aims to measure production readiness of a machine learning system by offering a scoring system that focuses on assessing testing and monitoring needs
Model Card	Aims to encourage transparent model reporting, clarifying the intended use cases of models and detailing performance characteristics
Standard Protocol Items: Recommendations for Interventional Trials (SPIRIT-AI)	Aims to promote transparent prospective evaluation and completeness of clinical trial protocol reporting for AI interventions; focuses on publishing the clinical trial protocol before the trial is conducted
Transparent Reporting of a multivariable prediction model for Individual Prognosis or Diagnosis (TRIPOD)	Aims to improve the transparency and reporting of studies developing, validating, or improving a prediction model

AI, artificial intelligence.

The AImedReport, conceptualized in 2022, was designed in concert with the AI Evaluation Framework by Overgaard et al^1^ in 2022, which outlines clinical AI research and development stages. This alignment was established to support compliance with reporting standards and provide a reference for the entire AI development lifecycle, aiding in informing development phases, engaging stakeholders, and supporting interpretability, knowledge continuity, transparency, and trust. While based on the framework by Overgaard et al,^1^ AImedReport’s matured versatility allows it to potentially suit other frameworks such as the SALIENT framework of van der Vegt et al^18^ for broader AI implementation.

Each reporting item was mapped to one of the phases of the AI Evaluation Framework^1^ to streamline documentation when knowledge is generated: prepare, develop, validate, deploy, and maintain. The “prepare” phase focuses on metadata related to the owner, defining the model’s purpose and clinical impact, data preparation, and planning for model development. The “develop” phase centers around model development and evaluation, usability related to inputs, assessing risk and bias, and protocol development for validation studies. The “validate” phase catalogs information about the design and execution of how the model was validated, summative usability testing, generating user education, and planning for deployment. The “deploy” phase focuses on clinical validation and generating training materials. Finally, the “maintain” phase plans for postdeployment surveillance and maintenance and quality monitoring and auditing. Reporting items were grouped into each of these 5 phases and then further classified into subgroups by identifying common themes (ie, prepare—purpose and clinical impact; develop—model development and evaluation; and deploy—clinical validation). For each reporting item, the team or team members that need to be involved at each phase and in what ways (eg, reporting, maintaining documentation, or utilization) were also defined (Table 2).

**Table 2:** AI Lifecycle Phases Into Which AImedReport “Reporting Items” Were Sorted, Along With Subphases and Interdisciplinary Alignment That Came From Further Organization

Research and discover		Translation	Deployment	
Prepare	Develop	Validate	Deploy	Maintain
Purpose and clinical impact Data preparation	Model development and evaluation Usability formative Model bias evaluation Study protocol development	Validation planning Usability summative User education Deployment planning	Clinical validation User training	Postdeployment surveillance and maintenance Quality monitoring and audit
Project manager Clinical expert UX researcher Ethicist Data engineer Data scientist Regulatory and legal Informaticist	Project manager Clinical expert UX researcher Ethicist Data engineer Data scientist Informaticist System engineer Software engineer	Project manager Clinical expert Ethicist Data engineer Data scientist System engineer UX researcher	Project manager Clinical expert Data scientist MLOps System engineer Software engineer UX researcher	Clinical expert Quality management MLOps Ethicist Informaticist Software engineer

MLOps, machine learning operation; UX, user experience.

## Discussion

The interactions among AI technologies, their users, and the implementation environments actively define the overall potential effectiveness of AI interventions within health care, especially because these tools are complex interventions designed as clinical decision support systems, not autonomous agents.^8^^,^^19^^,^^20^ A tailored, step-by-step approach may support the transition of AI technologies from being evaluated by statistical performance to clinical validity. To address this translational gap, AImedReport was developed to assist teams in several key areas, such as the following: (1) outlining phases of the AI lifecycle and clinical evaluation; (2) developing a comprehensive documentation deliverable and historical archive; and (3) addressing translation, implementation, and accountability gaps. This is achieved by consolidating the existing landscape of research reporting guidelines into a repository. This repository acts as a centralized documentation hub and provides a standardized list of considerations and accountability assignments as the solution advances across the development lifecycle. AImedReport (Appendix A), is presently a prototype tool housed in a spreadsheet but is planned to be made available as a web resource and a software platform. This will likely further enhance the tool’s usability, reproducibility, and convenience by providing the ability to automate the documentation process, enhance task completion and generate deliverables in accordance with relevant reporting measures, and allow for communication and updates to model documents to be centrally available across teams.

Introducing such a platform not only allows for transparent communication of evaluation and reporting measures but also embraces anticipated changes and modifications, which come with development and maintenance. Each reporting item can be assigned to a team or team member to define who is responsible, accountable, consulted, and informed, who can then use the reporting item description as a reference to satisfy their role.^21^ For example, project managers, user experience researchers, and machine learning operations can contribute model overview, goals, and future state from their respective perspectives and reference one another’s vision. Similarly, data scientists, AI ethicists, informatics teams, and clinical practice committees may use documented demographic data of patient populations to assess items such as bias, differential model performance, appropriate clinical location, and potential clinical workflow location. During deployment and maintenance, the primary user and updater of the documentation will be a machine learning operation team, to ensure requirements set by previous groups are met, monitoring input and output metrics for drift, volume, and appropriate use. This can also facilitate interoperability between organizations as the tool provides a standardized format, and documentation can be transferred across organizations and research governing bodies for consumption, auditing, and monitoring. AlmedReport also serves as a source of information describing completed evaluation and research reporting measures and can therefore fulfill reporting requirements to support clinical trial documentation and other publications. Additional descriptions of roles and responsibilities included within AImedReport, Appendix B.

This article focused on describing the theorization and development of AImedReport as a proof of concept to aid in evaluating, consolidating, and understanding available documentation resources to support AI reporting and facilitate communication across a multidisciplinary team. AlmedReport primarily concentrated on research reporting guidelines to address the immediate gaps identified within documentation practices. We note recent progress as the field rapidly advances toward enhancing implementation strategies within multidisciplinary teams. For example, in a study conducted by van der Vegt et al,^18^ an extensive mapping exercise was conducted to synchronize various guidelines with an AI implementation framework. We suggest that AImedReport could further contribute to such implementation endeavors as a valuable resource. Planned future work will continue to converge with and align to various frameworks, like the SALIENT framework,^18^ ABCDS,^22^ and organizations, such as the Office of the National Coordinator,^23^ Food and Drug Administration,^24^ Coalition for Health AI,^25^ National Academy of Medicine,^5^ Health AI Partnership,^26^ National Institute of Standards and Technology,^27^ and World Health Organization.^28^

We believe that AImedReport can be used in its current formative state for researchers and health care organizations to adhere to evaluation and research reporting standards as well as to bridge some of the reporting and documentation requirements for products necessitating design controls under good manufacturing practices of the quality system regulation, such as those that may be software as a Medical Device.^28^, ^29^, ^30^ Future iterations of AImedReport will better align translational science and regulatory science so that documentation can be used directly by teams pursuing regulated pathways, aligning with the information needed by regulatory review groups, accreditation commissions, and regulatory bodies (eg, Food and Drug Administration).

## Next Steps and Conclusion

Our multidisciplinary team developed AImedReport as a strategic effort to address collaboration and documentation challenges in AI translation. AImedReport functions to assist teams by (1) outlining phases of the AI lifecycle and clinical evaluation, (2) iteratively developing a comprehensive documentation deliverable and historical archive, and (3) addressing translation, implementation, and accountability gaps. By consolidating the existing landscape of research reporting guidelines into a repository, AImedReport acts as a centralized documentation hub that provides a standardized list of considerations and accountability assignments to guide information capture when knowledge is generated and simplify compliance with evaluation and reporting measures as AI technologies advances across the lifecycle. Completed measures documented within the AImedReport may also serve as a source of information to fulfill reporting requirements to support clinical trial documentation and other publications. The integration of AImedReport into existing IT infrastructure and reporting platforms has undergone phased development, starting with the creation of a Model Documentation Framework presented at the AMIA 2022 Clinical Informatics Conference, refined through feedback from the Coalition for Health AI in 2022,^31^ and forming the foundation for collaborative efforts across various AI evaluation considerations. Mayo Clinic’s regulatory and systems engineering teams are adapting the AImedReport framework to fit within regulatory infrastructure, aiming to scale multidisciplinary reporting for enterprise-wide AI applications. This integration process involves continued interdisciplinary collaboration and evaluation to ensure scalability and applicability across Mayo Clinic departments and disciplines.

Future work will include expanding AImedReport beyond a proof-of-concept phase and supporting various frameworks and organizations to enhance usability, including direct alignment of translational and regulatory sciences through FDA software as a Medical Device documentation.

## Potential Competing Interests

The authors report no competing interests.

## Footnotes

Supplemental material can be found online at https://www.mcpdigitalhealth.org/. Supplemental material attached to journal articles has not been edited, and the authors take responsibility for the accuracy of all data.
