# Supplementary material for: AImedReport: A Prototype Tool to Facilitate Research Reporting and Translation of Artificial Intelligence Technologies in Health Care
Source: Mayo Clin Proc Digit Health. 2024 Apr 6;2(2):246–51. doi: 10.1016/j.mcpdig.2024.03.008 (PMC11975813; doi:10.1016/j.mcpdig.2024.03.008)
Supplement: Supplementary Material [file mmc1.pdf]

Appendix

Appendix A: AImedReport

AImedReport is comprised of reporting items, outlined following product lifecycle phases to guide translation and promote transparent and explainable AI/ML-based MMS documentation. Access to the live document can be found here: **AImedReport Link**

Appendix B: Example AI Research Team Roles and Responsibilities

**Role**	**Example Accountability**
Project Manager	Manage the development and execution of a project, tracking progress and team communication to achieve project objectives.
Clinical Expert	Provides knowledge of clinical context throughout the AI lifecycle to inform design, usability, and adoption.
UX Researcher	Gathers user feedback and understands mental models of end users. Identifies users' needs and behaviors through direct observations, interviews, surveys, and usability evaluations to inform product design and development.
Ethicist	Identify types of possible harms, and specific potential subjects of harms; reason about underlying unjust historical and social processes that might eventually lead to differential model performance via measurement error, choice of sample, what data for which subjects are available or not.
Data Engineer	Manage data collection, connections, quality, cleaning, storage, processing and create data pipelines.
Data Scientist	Conduct data cleaning and quality checks, as well as employ appropriate statistical and machine learning approaches to uncover underlying meaningful relationships and patterns within datasets to allow for downstream actionable outcomes
MLOps	Actualize machine learning models into practice implementation and deployment through constructing necessary data connections and pipelines into production environments with appropriate model auditing, maintenance, monitoring, and feedback mechanisms in place.

System Engineer	Plan, define, test, and evaluate products for safety and efficacy against established best practices and procedures and external guidance or standards
Software Engineer	Build out data pipelines towards front-end users, accounting for resilient, scaled, and accurate cloud services through collaboration with data engineers and project developers to capture requirements and algorithms within application end products
Translational Informaticists	Bridges AI-model development and clinical implementation by aligning scientific and operational principles across teams
Regulatory and Legal	Manage and strategize compliance with regulatory policies and procedures, including those related to device classification and risk assessments.
Quality Management	Define and establish internal processes to fulfill quality mandates driven by institutions or external governance bodies.
